# Middle-range theory of the risk for ineffective glucose pattern self-management in hemodialysis

**DOI:** 10.1590/0034-7167-2024-0548

**Published:** 2026-02-23

**Authors:** Larissa de Lima Ferreira, Cláudio César Guimarães Martins, Lívia Hilário de Sousa Nunes, Annaiza Freitas Lopes de Araújo, Maria Isabel da Conceição Dias Fernandes, Ana Luisa Brandão de Carvalho Lira

**Affiliations:** I Universidade Federal do Rio Grande do Norte. Natal, Rio Grande do Norte, Brazil.; II Universidade Federal do Ceará. Fortaleza, Ceará, Brazil.

**Keywords:** Nursing Diagnosis, Blood Glucose, Hemodialysis, Nursing Theory, Adults

## Abstract

**Objectives:**

to develop a middle-range theory for the “Risk for ineffective glucose pattern self-management” nursing diagnosis in patients undergoing hemodialysis.

**Methods:**

this is a methodological study to validate the nursing diagnosis, based on a middle-range theory, conducted in six stages: defining the approach, theoretical-conceptual models, and concepts, developing the pictogram, propositions, and causal relationships. The construction was operationalized through a scoping review and supported by Roy’s model.

**Results:**

two essential attributes were identified, as well as 18 clinical antecedents, subdivided into seven risk factors, seven associated conditions, and four at-risk populations. A pictogram, six theoretical propositions, and causal relationships were identified in linear, domino, and qualitative leap effects.

**Conclusions:**

the middle-range theory of the “Risk for ineffective glucose pattern self-management” nursing diagnosis in hemodialysis patients was developed, helping to better understand this phenomenon.

## INTRODUCTION

Hemodialysis (HD) is one of the main renal replacement therapies (RRTs), characterized as a vital procedure for patients with acute and chronic renal failure^(1)^. This treatment aims to restore balance to the physiological system, remove excess fluids and undesirable solutes, and correct electrolyte and acid-base disturbances. However, it triggers a series of physiological changes during its implementation^(1)^, notably complications related to blood glucose, a phenomenon that can negatively impact the lives of patients requiring this therapy^(2)^.

It is known that renal failure and HD form a scenario prone to glycemic fluctuations^(2)^, which demands special attention from healthcare professionals, especially the nursing team, responsible for acting in the uninterrupted observation of patients during the HD period and which plays an important role in the prevention and detection of complications, such as hemodynamic changes, during treatment^(3)^.

Blood glucose fluctuations during HD are multifactorial and influenced by changes in glycemic homeostasis, insulin resistance, fluctuations in insulin levels, and glucose removal during blood filtration. This instability can result in hypoglycemia or hyperglycemia, both associated with adverse complications for patients^(4,5)^.

Therefore, glycemic instability is harmful to health, leading to worse outcomes, and can trigger serious consequences, such as increased susceptibility to infections, hydroelectrolytic disorders, endothelial dysfunction and thrombotic phenomena^(6)^. Therefore, it is necessary for nurses to identify and monitor complications during HD sessions, both to reduce occurrences and to reverse the risks of greater harm to patients^(7)^.

To this end, the application of the Nursing Process (NP) in the provision of quality nursing care is essential^(7)^. In this context, during the second stage of NP, the “Risk for ineffective glucose pattern self-management (00489)” nursing diagnosis (ND) can be listed in these patients. This ND is defined as the susceptibility to unsatisfactory management of symptoms, therapeutic regimen, and lifestyle changes associated with living with recurrent fluctuations in blood glucose levels outside the desired range^(8)^.

Therefore, studies that promote nursing care for renal patients on HD with fluctuating glucose levels are imperative. Understanding the phenomenon of blood glucose instability during HD allows nurses to exercise clinical judgment to develop a therapeutic plan tailored to patients’ specific needs^(9)^. To this end, the understanding of a nursing phenomenon can be achieved using middle-range theories (MRTs).

These theories focus on specific phenomena within the nursing context, serving as a strategy to bridge the gaps between theory, research, and practice. In nursing, an MRT is defined as a set of related ideas focused on a specific dimension of a phenomenon, including a limited number of concepts and propositions, described at a concrete level^(10)^.

To date, MRTs related to the NDs “Risk for excess fluid volume”^(11)^, “Sedentary lifestyle”^(12)^, “Risk for unstable blood pressure”^(13)^, “Overweight”^(14)^, “Ineffective peripheral tissue perfusion”^(15)^, “Risk for disturbed maternal-fetal dyad”^(16)^, and “Ineffective breathing pattern”^(17)^ have been found in the literature. In the context of nephrology, only one MRT was found focused on the existential dimension of being-in-the-world of chronic kidney disease^(18)^. Regarding glycemic alterations, a theoretical study with construct analysis and a proposal for the “Risk for imbalanced glycemic pattern” ND in adults with Diabetes Mellitus (DM)^(19)^ was identified. However, no MRT on the “Risk for ineffective glucose pattern self-management” ND in patients undergoing HD has been identified in the literature to date.

Therefore, it becomes relevant to construct an MRT of this phenomenon of interest to advance knowledge, through more specific concepts that can contribute to clinical nursing practice in favor of improving health conditions and patient safety in HD.

## OBJECTIVES

To develop an MRT of the “Risk for ineffective glucose pattern self-management” ND in patients undergoing HD.

## METHODS

### Ethical aspects

This research is theoretical in nature, and because it works with public domain studies, assessment by a Research Ethics Committee was not necessary.

### Study design

**Figure 1:**
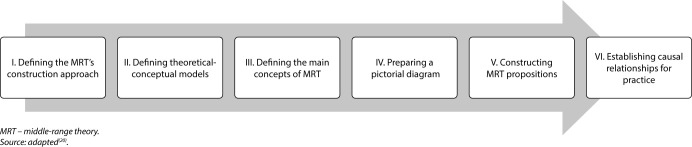
Study phases, Natal, Rio Grande do Norte, Brazil, 2024

Source: adapted^(20)^.

This is a methodological study of theoretical-causal validation of the “Risk for ineffective glucose pattern self-management” ND in adult and older patients undergoing HD, which was carried out in six stages^(20)^, as shown in Figure 1.

This type of research involves developing an MRT focused on identifying the etiological factors and defining characteristics of a ND, as well as verifying causal relationships that clarify the occurrence of this human response. It is noteworthy that, because this is a risk ND, defining characteristics were not identified.

In the first stage of this study, the objective of constructing the MRT for the “Risk for ineffective glucose pattern self-management” ND in patients undergoing HD was to establish precise relationships between concepts and describe how changes occur within a phenomenon. Thus, according to the literature^(20)^, these characteristics are present in predictive theory. Therefore, this MRT is predictive, but its orientation is deductive, as it was constructed from the synthesis of studies published in the literature on the phenomenon of interest^(20)^, identified through the construction of a scoping review.

Concerning the stage of defining the theoretical-conceptual models to be analyzed, resulting in the process of developing the MRT structure, the use of the theoretical model proposed by Roy was defined, which considers individuals as a holistic system, capable of adapting and promoting changes according to the environment in which they live^(21)^.

The Roy Adaptation Model (RAM) focuses on the interrelationship between the environment and the human adaptive system, categorized as a major nursing theory based on the interactive process. In this model, individuals, considered a system, can be influenced by the surrounding environment or by internal stimuli. These factors encompass all circumstances that can influence behavior and are categorized as focal, contextual, and residual stimuli. It is emphasized that the interaction of these factors is essential for the development of individuals’ level of adaptation, resulting in adaptive or ineffective responses^(21)^.

Focal stimuli are those stimuli, internal or external, that immediately confront a person. Contextual stimuli are all other stimuli present in the situation that contribute to the effect of the focal stimulus. In other words, all internal or external environmental factors that present themselves to a person, and even if not the center of attention, can influence how a person reacts to the focal stimuli. Residual stimuli, on the other hand, are internal or external environmental factors whose effects on the situation are unclear or simply cannot be assessed^(21)^.

To this end, the MRT was developed through a scoping review, which was developed based on the method proposed by the JBI Reviewer’s Manual (2020)^(22)^ and guided by the Preferred Reporting Items for Systematic reviews and Meta-Analyses extension for Scoping Reviews (PRISMA-ScR): Checklist and Explanation 18 recommendations^(23)^. The research protocol was registered in the Open Science Framework and is available at the link: DOI 10.17605/OSF.IO/EGZFS.

### Study protocol

The following descriptors indexed in the Medical Subject Headings/Health Sciences Descriptors were used for the search: “Patients/*Pacientes*” AND “Kidney Diseases/*Doenças Renais*”, “Blood Glucose/*Glicemia*” AND “Renal Dialysis/*Diálise Renal*”. The keywords “Hypoglycemia/*Hipoglicemia*”, “Hyperglycemia/*Hiperglicemia*”, “Glycemic Fluctuation/*Flutuação da Glicemia*” AND “Hemodialysis/Hemodiálise/*Diálise Extracorpórea*” were also used. Furthermore, the Boolean operators “AND” and “OR” were used.

The studies were searched for in peer-reviewed literature in the National Library of Medicine, COCHRANE, Web of Science, Scopus, and Latin American and Caribbean Literature in Health Sciences databases, and the Virtual Health Library. Gray literature was also searched through the databases Google Scholar, the Coordination for the Improvement of Higher Education Personnel Thesis and Dissertation Catalog, The National Library of Australia’s Trobe, Academic Archive Online, Europe E-Theses Portal, Electronic Theses Online Service, Open Access Scientific Repository of Portugal, Theses Canada, and Theses and Dissertations of Latin America.

Furthermore, a search was carried out in specific nephrology journals, with the following journals being selected: *Enfermería Nefrológica*, Brazilian Journal of Nephrology, Clinical Journal of the American Society of Nephrology and Nephrology Nursing Journal, but the latter was excluded due to all published works being unavailable for free search.

As for eligibility criteria, publications that met the study objective and were available in full were included. Studies in the form of editorials, letters to the editor, and opinion pieces were excluded. No language or time frame was defined, as the objective was to draw a timeline capable of recovering the largest number of studies addressing the subject of study.

### Data analysis

After carrying out the scoping review, the theoretical material was analyzed and, from this analysis, the main components for the construction of the MRT for the “Risk for ineffective glucose pattern self-management” ND in patients undergoing HD were extracted, such as essential attributes and risk factors (clinical antecedent), according to the reference^(11)^. Furthermore, clinical antecedents were categorized according to MAR into focal, contextual, and residual stimuli^(21)^.

The concepts found were then defined conceptually and operationally. Furthermore, to facilitate understanding of the theory in its specific context—i.e., how clinical antecedents and essential attributes relate to the study population—a pictogram was constructed^(20)^.

Furthermore, theoretical propositions were constructed to represent the interrelationships between concepts and the explanatory relationships between previously defined concepts. Causal relationships were also established to highlight the relationships between risk factors (antecedents) and effects when considering the ND as a product of nurses’ clinical reasoning^(20)^.

## RESULTS

The MRT for the “Risk for ineffective glucose pattern self-management” ND in patients undergoing HD was developed to establish precise relationships between concepts and describe how changes occur within this phenomenon. According to the literature^(20)^, these characteristics are present in predictive theory. Thus, this theory is predictive, but its orientation is deductive, as it was constructed from the synthesis of available studies in the literature, identified through a scoping review. The results will be presented according to the stages for constructing the MRT.

### Defining the middle-range theory’s construction approach

**Figure 2:**
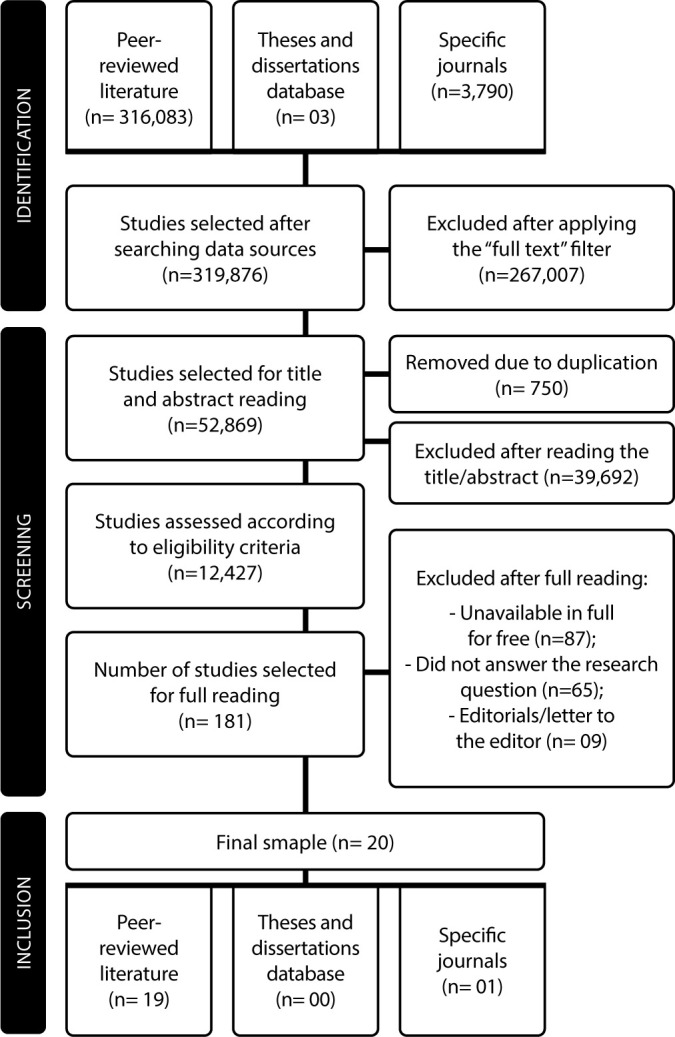
Literature search flowchart adapted from the Preferred Reporting Items for Systematic reviews and Meta-Analyses extension for Scoping Reviews, Natal, Rio Grande do Norte, Brazil, 2024

In stage I, it was defined that the MRT would be developed based on the synthesis of published research on the phenomenon of interest. Thus, the final sample consisted of 20 studies, and the follow-up of this entire process is displayed in the PRISMA-ScR flowchart and presented in Figure 2.

### Defining the middle-range theory’s theoretical-conceptual models and main concepts

Based on the articles selected by the scoping review, it was possible to list the essential attributes and clinical antecedents of risk for unstable blood glucose levels, as well as develop their conceptual and operational definitions, in order to clarify their clinical significance. As previously mentioned, clinical antecedents were categorized according to the MAR into focal, contextual, and residual stimuli^(21)^.

To develop the MRT, two essential attributes were used: HD and glycemic variation outside the normal range. Thus, the diagnosis under study was defined as “susceptibility to glycemic variation outside the normal range during and/or after HD”.

Furthermore, from the scoping review, 18 clinical antecedents were identified, which were classified, according to the literature^(24)^, into causal etiological factors, namely: predisposing factors, which refer to those that increase susceptibility to ND; disabling factors, which are those that interfere with health recovery or promotion; precipitating factors, which represent those that initiate the causal chain; and reinforcing factors, which are capable of amplifying the effect of an existing condition, as shown in Chart 1.

**Chart 1:** Classification of clinical antecedents into predisposing, disabling, precipitating, and reinforcing etiological factors, Natal, Rio Grande do Norte, Brazil, 2024

ETIOLOGICAL FACTORS	ANTECEDENTS
**Predisposing factors**	Weight loss; high Body Mass Index; Diabetes Mellitus; insulin resistance; malnutrition; hyperlipidemia; and infections.
**Disabling factors**	Inadequate blood glucose monitoring during and/or after hemodialysis; inadequate knowledge about medication regimen; inadequate knowledge about signs and symptoms of hypoglycemia; older adults; uremic individuals; hospitalized individuals; and individuals with a longer time since diagnosis of Diabetes Mellitus.
**Precipitant factors**	Glucose-free dialysate; and high-flux dialyzer filter.
**Reinforcing factors**	Inadequate food intake; and total fasting.

Source: adapted^(24)^.

Clinical antecedents were also subdivided into risk factors, associated conditions, and population at risk, which were listed in Table 1.

**Table 1:** Risk factors, associated conditions, and population at risk for the “Risk for unstable blood glucose level” nursing diagnosis in hemodialysis patients, Natal, Rio Grande do Norte, Brazil, 2024

Risk factors	n*	%
Inadequate blood glucose monitoring during and/or after hemodialysis	09	32.2
Inadequate knowledge of medication regimen	08	28.6
Inadequate dietary intake	05	17.8
Weight loss	01	03.6
Inadequate knowledge of signs and symptoms of hypoglycemia	01	03.6
High Body Mass Index	01	02.7
Total fasting	01	03.6
**Associated conditions**	**N**	**%**
Diabetes Mellitus	19	51.3
Glucose-free dialysate	09	24.3
Insulin resistance	04	10.8
Malnutrition	03	10.7
High-flux dialyzer filter	03	08.1
Hyperlipidemia	01	02.7
Infections	01	02.7
**Population at risk**	**N**	**%**
Older adults	04	44.4
Individuals with a longer history of Diabetes Mellitus diagnosis	01	11.1
Uremic individuals	01	11.1
Hospitalized individuals	01	11.1

*More than one of the antecedents were present in the same study, which justifies the sum exceeding 100.0%.

Thus, seven risk factors, seven associated conditions and four populations at risk for the “Risk for ineffective glucose pattern self-management” ND in patients undergoing HD were identified and were categorized according to the RAM into focal, contextual, and residual stimuli.

Thus, the risk factors inadequate dietary intake, weight loss, and total fasting were identified as focal stimuli. Contextual stimuli were identified in the following risk factors: inadequate blood glucose monitoring during and/or after HD; inadequate knowledge about medication regimen; inadequate knowledge about signs and symptoms of hypoglycemia; high body mass index; associated conditions: DM; glucose-free dialysate; insulin resistance; malnutrition; high-flux dialyzer filter; hyperlipidemia; infections; and in at-risk populations: uremic and hospitalized individuals. Residual stimuli were identified in at-risk populations, namely, older adults and individuals with a longer time since DM diagnosis.

### Preparation of a pictorial diagram

The MRT developed in this study was entitled “Nursing theory for ‘Risk for ineffective glucose pattern self-management’ in patients undergoing HD”, and from the grouping of the identified elements, it was possible to construct a pictogram capable of synthesizing the theory.

**Figure 3:**
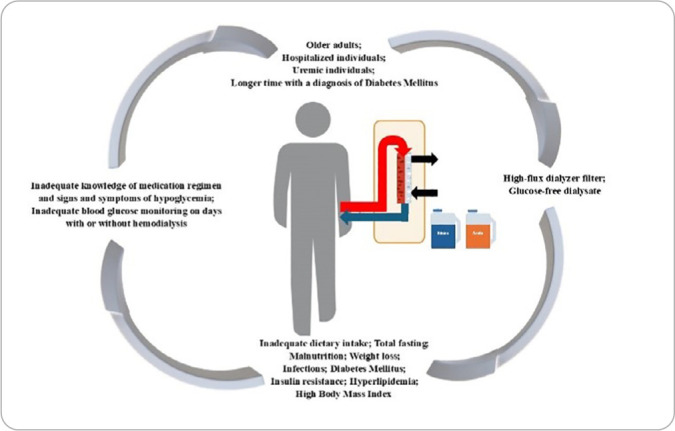
Pictogram showing the main interrelated concepts of the “Risk for ineffective glucose pattern self-management” theory in adult and older patients undergoing hemodialysis, Natal, Rio Grande do Norte, Brazil, 2024

Figure 3 presents the pictogram constructed for the theory and its elements, explaining the relationships between the concepts involving the risk for ineffective glucose pattern self-management in adult and older patients undergoing HD.

Thus, the image of an human being represents the individual undergoing HD with a greater risk of blood glucose variation connected, from the vascular access, dialyzer (site of interaction of the blood and dialysis solution circuits and where the movement of molecules between the dialysis solution and the blood occurs through a semipermeable membrane), and efflux equipment (which conducts the blood from the dialyzer back to the vascular access).

Thus, to explain the risk for ineffective glucose pattern self-management in patients undergoing HD, it is observed that it is influenced by various stimuli that can trigger ineffective behavior, such as episodes of hypo/hyperglycemia during and after treatment. These stimuli (focal, contextual, and residual) are represented in the image by arrows in the same hue as the individuals’ image. They directly and indirectly influence patients undergoing HD and are responsible for increasing this patient’s risk of developing blood glucose fluctuations during and after treatment.

Thus, factors that contribute to vulnerability to glycemic imbalance are observed, and these interact with each other and may be subject to independent intervention by the nursing team. Furthermore, the cyclical arrows indicate that stimuli can influence individuals with varying degrees of intensity.

### Constructing the middle-range theory propositions

For the MRT developed in this study, six propositions were created, which provide the causal relationships between the clinical antecedents and essential attributes of risk for ineffective glucose pattern self-management in adult/older patients undergoing HD.

1. The glucose variation of patients undergoing HD is affected by stimuli originating from the patient (age, comorbidities and ineffective self-management) and influenced by the actions of healthcare professionals involved in HD (provision of food, administration of antiglycemic agents before HD, monitoring of blood glucose during and after HD, prescription of treatment with high-flux dialyzers, and glucose-free dialysate);
2. Individuals who are completely fasting or have inadequate food intake when undergoing HD have a greater risk for unstable blood glucose levels;
3. Weight loss, malnutrition, insulin resistance, hyperlipidemia, DM, infections, and high Body Mass Index in patients undergoing HD increase the risk for unstable blood glucose levels;
4. Individuals with a longer time since DM diagnosis, older adults, with uremia and/or hospitalized, when undergoing HD, may be susceptible to the risk of unstable blood glucose levels;
5. Patients undergoing HD’ lack of knowledge about the signs and symptoms of hypoglycemia and the medication regimen increases the risk of unstable blood glucose levels;
6. Failure to monitor patients’ glucose and inadequate monitoring may make it difficult to identify HD-induced glycemic disturbance.

### Establishing causal relationships for practice

Patients undergoing HD have a higher risk for ineffective glucose pattern self-management, with episodes of hypoglycemia attributed to several factors, which include, due to kidney disease, decreased renal gluconeogenesis, impaired insulin clearance by the kidneys, deficient insulin degradation due to uremia, increased glucose uptake by erythrocytes during HD, impaired counter-regulatory hormonal responses, inadequate dietary intake, use of oral antidiabetic medications or exogenous insulin without adequate monitoring of blood glucose levels, effects of dialysis treatment, and even age or hospitalization^(1,4,5)^.

Furthermore, since insulin secretion can be stimulated by dialysate glucose, patients with moderate levels of β-cell function are at increased risk of hypoglycemia with HD, particularly after experiencing large hyperglycemic excursions. Conversely, patients with decreased or nonexistent β-cell function are at increased risk of postprandial hyperglycemia after HD due to increased insulin resistance, insulin adsorption by dialyzers, and secretion of counterregulatory hormones in response to the hypoglycemic state^(1,4,5)^.

According to the literature^(20)^, causal relationships can be of seven different types: linear effect; trigger effect; feedback effect; domino effect; butterfly effect; quality leap effect; and effect of sufficient causes. Thus, the cause-and-effect relationships for the risk for ineffective glucose pattern self-management in patients undergoing HD were established:

- Linear effect: inadequate food intake; total fasting; glucose-free dialysate; high-flux dialysis filter. Thus, exposure to the antecedent generates a consequence, which is ineffective self-management of blood glucose levels;
- Domino effect: inadequate blood glucose monitoring during and/or after HD; inadequate knowledge of medication regimen; inadequate knowledge about signs and symptoms of hypoglycemia. In these cases, a precedent generates a consequence that leads to a subsequent change;
- Quality leap effect: high Body Mass Index; DM; insulin resistance; malnutrition; hyperlipidemia; infections. In these situations, constant exposure to a clinical antecedent can lead to more serious consequences.

Therefore, given the above, managing blood glucose levels in patients undergoing HD is a complex process. Causal relationships and evidence for practice provide the theoretical basis for ND validation and clinical nursing practice.

## DISCUSSION

To make an accurate diagnosis, nurses must have a thorough understanding of key concepts related to NDs. Furthermore, they must accurately interpret human responses to select appropriate interventions and assess outcomes^(25)^. In this context, the NANDA-I taxonomy enables a standardized language for NDs, which is essential to promote clarity and consistency in communication among nursing professionals, in addition to strengthening their practice and promoting consistent care planning^(8)^.

Furthermore, this uniformity in language contributes to nursing research, allowing for the comparison and analysis of data across different contexts and geographic regions, which adds information and sparks discussions within the nursing field. Thus, it fosters the development and refinement of NDs, making their language more robust, standardized, and evidence-based^(8)^.

Therefore, in relation to NANDA-I taxonomy improvement, the present study enabled the development of a nursing theory for the “Risk for ineffective glucose pattern self-management” ND in patients undergoing HD, and the scoping review allowed the understanding of a theoretical gradient on the phenomenon studied.

Furthermore, there was a predominance of productions in article format, which may be associated with the speed of preparation and dissemination of this format, in addition to having greater visibility, compared to dissertations and theses^(26)^. Despite the notable importance of the topic in the study population, a small number of studies addressing the phenomenon of unstable glycemia in patients undergoing HD were found, with a predominance of studies on the topic in the United States. This result may reflect the significant growth in the number of DM cases, the main pathology leading to end-stage renal disease and the need for HD^(27)^.

However, there has been an increase in publications over the past five years, a fact that reflects the relevance of the topic, since changes in glucose levels are concerning and can trigger severe complications. Both spikes in blood glucose levels and their abrupt decreases should be avoided and controlled to improve the quality of therapy, prognosis, and patient safety^(2)^.

It is important to highlight that the review did not find any studies that addressed the “Risk for ineffective glucose pattern self-management” ND in patients undergoing HD and the development of an MRT. Concerning available evidence-based studies/guidelines for glycemic targets in patients undergoing HD, the literature^(27)^ recommends that patients with DM and dialysis use insulin regimens based on HD time and pre- and post-dialysis blood glucose levels, requiring a reduction of at least 25% in the dose of rapid or ultra-rapid insulin administered immediately before the meal prior to dialysis, in addition to maintaining blood glucose levels between 126 and 200 mg/dL before and during HD.

Furthermore, when comparing the elements of the “Risk for ineffective glucose pattern self-management” ND present in NANDA-I^(8)^, which currently has 28 risk factors, 23 associated conditions and 13 populations at risk, based on the scoping review, this ND in patients undergoing HD presented seven risk factors, and among them, inadequate knowledge about signs and symptoms of hypoglycemia stands out.

It is important to emphasize that patients need to be aware of the signs and symptoms of glycemic lability, as lack of knowledge can lead to hypoglycemia being misdiagnosed as hypovolemia, imbalance due to HD, or electrolyte imbalances. It is important to note that hypoglycemia symptoms are divided into autonomic symptoms and neurological hypoglycemia symptoms. Autonomic nervous system symptoms include hunger, sweating, anxiety, paresthesia, palpitations, tremors, pallor, and tachycardia. Neurological hypoglycemia symptoms include weakness, dizziness and headache, confusion, abnormal behavior, cognitive impairment, blurred vision, diplopia, central blindness, hypothermia, epilepsy, and coma^(28,29)^.

Regarding associated conditions, only seven were listed, with two notable ones: glucose-free dialysate and high-flux dialyzer filter. Dialysate is the dialysis solution, which must be prepared with purified (treated) water and concentrates (electrolytes necessary to obtain the prescribed composition of the dialysis solution). Therefore, a glucose-free dialysate can induce losses of up to 30 g of glucose per session due to glucose loss through the capillary membranes, which can lead to hypoglycemia^(1,30)^.

The dialysis filter is the site of interaction between the blood and dialysis solution circuits, i.e., where the movement of molecules between the dialysis solution and the blood occurs through a semipermeable membrane^(1)^. Thus, the removal of substances is influenced by factors such as reflection coefficient, pore structure, hydraulic permeability, dialysis membrane surface, and dialysis filter fiber thickness^(31)^. Therefore, membranes with high water permeability and large pores (high flux) have very low resistance and facilitate the diffusion of molecules, which subjects patients to a greater risk of glucose loss during treatment^(32)^.

The populations undergoing HD at risk for unstable blood glucose levels identified were adults and older patients with a longer DM diagnosis, uremic patients, and hospitalized patients. It is noteworthy that individuals with a previous DM diagnosis and undergoing HD are at greater risk for unstable blood glucose levels, as they may be more accustomed to a less rigid treatment regimen and, therefore, less likely to change and more exposed to risk^(33)^.

As for uremic patients, they present signs and symptoms, such as nausea, vomiting, decreased level of consciousness and disorientation, which can increase the risk for ineffective glucose pattern self-management, not only due to the lack of food and nutrient intake, but also due to the need for strict diets with protein and calorie restriction^(34)^.

Furthermore, glycemic instability in hospitalized patients can occur due to stressful situations (stress hyperglycemia) and due to a lack of prior knowledge of DM diagnosis^(35)^. Changes in diet due to hospitalization are also common, such as the need for prolonged fasting and non-compliance with the diet offered. Furthermore, hospitalized patients receiving enteral nutrition may be susceptible to unscheduled interruptions in enteral or parenteral nutrition. Furthermore, during hospitalization, medications such as corticosteroids, which can alter blood glucose levels, may be necessary^(36)^.

In this context, with the observation of the high incidence of variation in blood glucose in patients undergoing HD, it is evident that those who require this therapy are more susceptible to instability in glucose levels due to renal failure itself, which predisposes to disorders in glucose homeostasis in individuals with and/or without DM, since the kidney plays a crucial role in glucose and insulin metabolism^(1,5,37)^.

Therefore, it is imperative that nursing interventions are conducted based on nurses’ clinical judgment and theoretical knowledge^(23)^, for better outcomes for patients undergoing HD, since these interventions have the potential to improve glycemic control and, consequently, reduce associated complications and mortality.

### Study limitations

Among the study’s limitations, it is worth noting that this MRT was developed for adult and older patients undergoing HD. Therefore, generalizing these results to pediatric patients and other types of RRT should be done with caution. Another limitation was the extraction of key concepts, essential attributes, and clinical antecedents, which was performed solely by the principal investigator, without the assistance of other researchers and/or software.

### Contribution to health, nursing or public policy

This study aims to broaden discussions in the scientific and healthcare community about the vulnerability of patients undergoing HD to changes in blood glucose levels during treatment. It also contributes to the improvement of the “Risk for ineffective glucose pattern self-management” ND, which has not yet been validated for patients undergoing HD. This can support the development of strategies and the reorganization of nursing work to minimize blood glucose instability and its effects on patients requiring this treatment.

## CONCLUSIONS

The theoretical development identified two essential attributes: HD and glycemic variation outside the normal range. Eighteen clinical antecedents were also identified. These were classified into predisposing, disabling, precipitating, and reinforcing etiological factors, subdivided into seven risk factors, seven associated conditions, and four at-risk populations. Furthermore, a pictogram, six propositions, and causal relationships were developed, and practical evidence arising from the interrelationships between these concepts was classified into linear, domino, and qualitative leap effects.

Thus, the MRT for the “Risk for ineffective glucose pattern self-management” ND in patients undergoing HD was developed, contributing to a greater understanding of this phenomenon in the scientific and healthcare community. Further studies are suggested to empirically validate this MRT in the adult population undergoing HD.

## Data Availability

The research data are available within the article.
