# Supplementary material for: Middle-range theory of the risk for ineffective glucose pattern self-management in hemodialysis
Source: Rev Bras Enferm. 2026 Feb 23;79:e20240548. doi: 10.1590/0034-7167-2024-0548 (PMC12933934; doi:10.1590/0034-7167-2024-0548)
Supplement: Translation

# Translation: Teoria de médio alcance do risco de autogestão ineficaz do padrão de glicemia na hemodiálise

**DOI:** 10.1590/0034-7167-2024-0548pt

**Authors:** Larissa de Lima Ferreira, Cláudio César Guimarães Martins, Lívia Hilário de Sousa Nunes, Annaiza Freitas Lopes de Araújo, Maria Isabel da Conceição Dias Fernandes, Ana Luisa Brandão de Carvalho Lira

**Affiliations:** I Universidade Federal do Rio Grande do Norte. Natal, Rio Grande do Norte, Brasil.; II Universidade Federal do Ceará. Fortaleza, Ceará, Brasil.

**Keywords:** Diagnóstico de Enfermagem, Glicemia, Hemodiálise, Teoria de Enfermagem, Adultos

## Abstract

**Objetivos:**

elaborar uma teoria de média alcance do diagnóstico de enfermagem “Risco de autogestão ineficaz do padrão de glicemia” em pacientes submetidos à hemodiálise.

**Métodos:**

estudo metodológico de validação do diagnóstico de enfermagem, a partir de uma teoria de médio alcance, realizado em seis etapas: definição da abordagem, dos modelos teórico-conceituais e dos conceitos, elaboração do pictograma, das proposições e das relações de causalidade. A construção foi operacionalizada pela *scoping review* e subsidiada pelo modelo de Roy.

**Resultados:**

foram identificados dois atributos essenciais, 18 antecedentes clínicos, subdivididos em sete fatores de risco, sete condições associadas e quatro populações em risco, um pictograma, seis proposições teóricas, e as relações de causalidade em efeitos linear, dominó e salto de qualidade.

**Conclusões:**

a teoria de médio alcance do diagnóstico de enfermagem “Risco de autogestão ineficaz do padrão de glicemia” nos pacientes em hemodiálise foi elaborada, auxiliando na maior compreensão desse fenômeno.

## INTRODUÇÃO

A hemodiálise (HD) é uma das principais terapias renais substitutivas (TRSs), caracterizando-se como um procedimento vital para pacientes com insuficiência renal aguda e crônica^1^Esse tratamento tem como função recuperar o equilíbrio do sistema fisiológico, remover os líquidos em excesso, solutos indesejáveis, e corrigir os distúrbios eletrolíticos e acidobásico. Entretanto, desencadeiam uma série de alterações fisiológicas durante sua realização^1^, com destaque para complicações relacionadas à glicemia, um fenômeno que pode impactar negativamente a vida dos pacientes que necessitam dessa terapia^2^.

Sabe-se que a insuficiência renal e a HD formam um cenário propenso a flutuações glicêmicas^2^, o que demanda atenção especial dos profissionais de saúde, principalmente da equipe de enfermagem, responsável por atuar na observação ininterrupta dos pacientes no período hemodialítico e que possui importante papel na prevenção e constatação de complicações, como as alterações hemodinâmicas, durante o tratamento^3^.

A variação da glicemia durante a HD é multifatorial e influenciada por alterações na homeostase glicêmica, na insulinorresistência, nas variações dos níveis de insulina e na remoção de glicose durante o processo de filtração sanguínea. Essa instabilidade pode resultar em hipoglicemia ou hiperglicemia, ambas associadas a complicações adversas para o paciente^(4, 5^).

Destarte, a instabilidade glicêmica é deletéria à saúde, levando a piores desfechos, e pode desencadear consequências graves, como aumento na susceptibilidade a infecções, distúrbios hidroeletrolíticos, disfunção endotelial e fenômenos trombóticos^6^Por isso, é necessário que o enfermeiro identifique e monitore as intercorrências durante as sessões de HD, tanto para reduzir as ocorrências quanto para reverter os riscos de danos maiores aos pacientes^7^.

Para tanto, é primordial a aplicação do Processo de Enfermagem (PE) na prestação de uma assistência de enfermagem de qualidade^7^Nesse contexto, durante a segunda etapa do PE, pode-se elencar, nestes pacientes, o diagnóstico de enfermagem (DE) “Risco de autogestão ineficaz do padrão de glicemia (00489)”. Esse DE é definido como a suscetibilidade a manejo insatisfatório de sintomas, regime terapêutico e mudanças no estilo de vida associados à convivência com flutuações recorrentes no nível de glicose sanguínea fora da faixa desejável^8^.

Nesse sentido, torna-se imperativo estudos que fomentem o cuidado de enfermagem ao paciente renal em HD com variação do nível de glicose. Assim, compreender o fenômeno da instabilidade da glicemia durante a HD permite que o enfermeiro realize um julgamento clínico para desenvolver um plano terapêutico direcionado às reais necessidades desta clientela^9^Para tal, a compreensão de um fenômeno de enfermagem pode ser realizada a partir de teorias de médio alcance (TMAs).

Essas teorias concentram-se em fenômenos específicos dentro do contexto da enfermagem, sendo uma estratégia para diminuir as lacunas entre teoria, pesquisa e prática. Na enfermagem, uma TMA é definida como um conjunto de ideias relacionadas com foco em uma dimensão específica de um fenômeno, incluindo um número restrito de conceitos e proposições, descritos em um nível concreto^10^.

Foram encontradas na literatura, até o momento, TMAs relacionadas aos DEs “Risco de volume de líquidos excessivos”^11^, “Estilo de vida sedentário”^12^, “Risco de pressão arterial instável”^13^, “Sobrepeso”^14^, “Perfusão tissular periférica ineficaz”^15^, “Risco de binômio mãe-feto perturbado”^16^ e “Padrão respiratório ineficaz”^17^No contexto da nefrologia, encontrou-se apenas uma TMA voltada para a dimensão existencial do ser-no-mundo da doença renal crônica^18^Quanto às alterações glicêmicas, foi identificado estudo teórico com análise de construtos e proposta de DE “Risco para padrão glicêmico desequilibrado” em adultos com Diabetes Mellitus (DM)^19^Entretanto, nenhuma TMA sobre o diagnóstico “Risco de autogestão ineficaz do padrão de glicemia” em pacientes submetidos à HD foi identificada na literatura até o momento.

Dessa forma, torna-se relevante construir uma TMA desse fenômeno de interesse para avançar o conhecimento, por meio de conceitos mais específicos que poderão contribuir com a prática clínica de enfermagem em prol da melhoria das condições de saúde e da segurança do paciente em HD.

## Objetivos

Elaborar uma TMA do DE “Risco de autogestão ineficaz do padrão de glicemia” em pacientes submetidos à HD.

## MÉTODOS

### Aspectos éticos

Esta pesquisa é de natureza teórica, e por trabalhar com estudos de domínio público, a apreciação do Comitê de Ética em Pesquisa não foi necessária.

### Desenho do estudo

Trata-se de estudo metodológico de validação teórico-causal do DE “Risco de autogestão ineficaz do padrão de glicemia” em pacientes adultos e idosos submetidos à HD, o qual foi realizado em seis etapas^20^, conforme apresentado na Figura 1.

**Figura 1:** Fases do estudo, Natal, Rio Grande do Norte, Brasil, 2024

Este tipo de pesquisa consiste no desenvolvimento de uma TMA focada na identificação de fatores etiológicos e características definidoras de um DE, além de verificar relações causais que esclareçam a ocorrência dessa resposta humana. Destaca-se que, por se tratar de um DE de risco, não foram identificadas as características definidoras.

Na primeira etapa do presente estudo, o objetivo da construção da TMA para o DE “Risco de autogestão ineficaz do padrão de glicemia” em pacientes submetidos à HD foi estabelecer as relações precisas entre os conceitos e descrever como ocorrem mudanças dentro de um fenômeno. Assim, conforme a literatura^20^, essas características estão presentes na teoria preditiva. Portanto, a presente TMA é preditiva, mas sua orientação é dedutiva, pois foi construída a partir da síntese dos estudos publicados na literatura sobre o fenômeno de interesse^20^, identificados mediante a construção de *scoping review*.

Com relação à etapa de definição dos modelos teóricos-conceituais a serem analisados, resultando no processo de desenvolvimento da estrutura da TMA, definiu-se a utilização do modelo teórico proposto por Roy que considera o indivíduo como um sistema holístico, apto a se adaptar e promover mudanças de acordo com o ambiente em que vive^21^.

O Modelo de Adaptação de Roy (MAR) é centrado na interrelação entre ambiente e sistema adaptativo humano, categorizado como uma grande teoria da enfermagem baseada no processo interativo. Nesse modelo, o indivíduo, considerado um sistema, pode ser influenciado pelo ambiente ao seu redor ou por estímulos internos. Esses fatores englobam todas as circunstâncias que podem influenciar o comportamento e são categorizados em estímulos focais, contextuais e residuais. Ressalta-se que a interação desses fatores é essencial para o desenvolvimento do nível de adaptação do indivíduo, resultando em respostas adaptativas ou ineficazes^21^.

Estímulos focais são aqueles estímulos, internos ou externos, que confrontam imediatamente a pessoa. Os estímulos contextuais são todos os outros estímulos presentes na situação e que contribuem para o efeito do estímulo focal, isto é, todos os fatores ambientais internos ou externos que se apresentam à pessoa, e mesmo não sendo o centro da atenção, podem influenciar a forma como a pessoa reage aos estímulos focais. Já os estímulos residuais são fatores ambientais internos ou externos, cujos efeitos na situação não se encontrem claros ou, simplesmente, não possam ser avaliados^21^.

Para tanto, a elaboração da TMA foi operacionalizada por meio de *scoping review*, a qual foi elaborada a partir do método proposto pelo JBI Reviewer’s Manual (2020)^22^ e orientada segundo as recomendações do Preferred Reporting Items for Systematic reviews and Meta-Analyses extension for Scoping Reviews (PRISMA-ScR): Checklist and Explanation 18^23^O protocolo de pesquisa foi registrado no *Open Science Framework*, e encontra-se disponível no *link*: DOI 10.17605/OSF.IO/EGZFS.

### Protocolo do estudo

Para a busca, foram utilizados os seguintes descritores indexados ao *Medical Subject Headings*/Descritores em Ciências da Saúde: “*Patients*/Pacientes” AND “*Kidney Diseases*/Doenças Renais”, “*Blood Glucose*/Glicemia” AND “*Renal Dialysis*/Diálise Renal”. E foram utilizadas as palavras-chave: “*Hypoglycemia*/Hipoglicemia”, “*Hyperglycemia*/Hiperglicemia”, “*Glycemic Fluctuation*/Flutuação da Glicemia” AND “*Hemodialysis*/Hemodiálise/Diálise Extracorpórea”. Além disso, utilizaram-se os operadores booleanos “*AND*” e “*OR*”.

Os estudos foram buscados na literatura revisada por pares nas bases de dados *National Library of Medicine*, COCHRANE, *Web of Science*, Scopus e Literatura Latino-Americana e do Caribe em Ciências da Saúde, Biblioteca Virtual em Saúde. Também foram buscados na literatura cinzenta através das bases de dados *Google Scholar*, Catálogo de Teses e Dissertações da Coordenação de Aperfeiçoamento de Pessoal de Nível Superior, *The National Library of Australia’s Trobe, Academic Archive Online, Europe E-Theses Portal, Electronic Theses Online Service*, Repositório Científico de Acesso Aberto de Portugal, Theses Canada e Teses e Dissertações da América Latina.

Além disso, foi realizada a busca em periódicos específicos da nefrologia, sendo selecionadas as revistas *Enfermería Nefrológica, Brazilian Journal of Nephrology, Clinical Journal of the American Society of Nephrology e Nephrology Nursing Journal*, mas essa última foi excluída devido a todos os trabalhos publicados estarem indisponíveis para busca de forma gratuita.

Quanto aos critérios de elegibilidade, foram incluídas publicações que respondessem ao objetivo de estudo e que estavam disponíveis na íntegra. Excluíram-se estudos em formato de editorias, carta ao editor e artigos de opinião. Não foram delimitados idioma, nem recorte temporal, pois objetivou-se traçar uma linha do tempo capaz de recuperar o maior número de pesquisas que abordassem o objeto de estudo.

### Análise dos dados

Após realização da *scoping review*, o material teórico foi analisado e, a partir dessa análise, foram extraídos os principais componentes para a construção da TMA para o DE “Risco de autogestão ineficaz do padrão de glicemia” em pacientes submetidos à HD, como atributos essenciais e fatores de risco (antecedentes clínicos), conforme referencial^11^Além disso, os antecedentes clínicos foram categorizados conforme o MAR em estímulos focais, contextuais e residuais^21^.

Em seguida, os conceitos encontrados foram definidos conceitualmente e operacionalmente. Além disso, para facilitar a compreensão da teoria em seu contexto específico, ou seja, como se relacionam os antecedentes clínicos e atributos essenciais na população estudada, foi construído o pictograma^20^.

Ademais, realizou-se a construção das proposições teóricas para representar as inter-relações entre os conceitos e as relações explicativas ente os conceitos previamente definidos. Também foram estabelecidas as relações de causalidades, a fim de evidenciar quais as relações existentes entre os fatores de risco (antecedentes) e os efeitos ao considerar o DE como produto do raciocínio clínico do enfermeiro^20^.

## RESULTADOS

Elaborou-se a TMA do DE “Risco de autogestão ineficaz do padrão de glicemia” em pacientes submetidos à HD com o objetivo de estabelecer as relações precisas entre os conceitos e descrever como ocorrem mudanças dentro desse fenômeno. Conforme a literatura^20^, essas características estão presentes na teoria preditiva. Assim, a presente teoria é preditiva, mas sua orientação é dedutiva, pois foi construída a partir da síntese dos estudos disponíveis na literatura, identificados mediante uma *scoping review*. Os resultados serão apresentados de acordo com as etapas para a construção da TMA.

### Definição de abordagem de construção da teoria de médio alcance

Na etapa I, definiu-se que a TMA seria desenvolvida a partir da síntese de pesquisas publicadas sobre o fenômeno de interesse. Dessa forma, a amostra final foi de 20 pesquisas, e o seguimento de todo esse processo está disposto no fluxograma PRISMA-ScR e apresentado na Figura 2.

**Figura 2:** Fluxograma de busca na literatura adaptado do Preferred Reporting Items for Systematic reviews and Meta-Analyses extension for Scoping Reviews, Natal, Rio Grande do Norte, Brasil, 2024

### Definição dos modelos teórico-conceituais e dos conceitos principais da teoria de médio alcance

A partir dos artigos selecionados pela *scoping review,* foi possível elencar os atributos essenciais e antecedentes clínicos do risco de glicemia instável, bem como elaborar suas definições conceituais e operacionais, de modo a esclarecer seu significado clínico. Conforme comentado anteriormente, os antecedentes clínicos foram categorizados conforme o MAR em estímulos focais, contextuais e residuais^21^.

Para a elaboração da TMA, foram utilizados dois atributos essenciais: HD e variação glicêmica fora do padrão de normalidade. Assim, construiu-se a definição do diagnóstico em estudo como “suscetibilidade à variação glicêmica fora do padrão de normalidade durante e/ou após a HD”.

Ademais, a partir da *scoping review,* foram identificados 18 antecedentes clínicos, que foram classificados, conforme a literatura^24^, em fatores etiológicos causais, sendo eles: fatores predisponentes, os quais se referem àqueles que aumentam a suscetibilidade ao DE; fatores incapacitantes, que são os que interferem na recuperação ou promoção da saúde; fatores precipitantes, que representam aqueles que iniciam a cadeia causal; e fatores de reforço, que são capazes de amplificar o efeito de uma condição existente, conforme apresentado no Quadro 1.

**Quadro 1:** Fatoros etiológicos e antecdentes

FATORS ETIÓLÓICAS	ANTECDENTES
**Predisponentes**	Perda de weso; Índice de Massa Corroral algo; Diabetus; Diabetes Mellittus; resistence à insulina; desnutrição, hiperlipéndia, e infreções.
**Incabiitantes** Monitorizção adequuade da glicemia durane e/00 apáls a hemodilalsie; conoclizendo indeibaçe indequão corr cone sinais e sintomas de hipoglicemia.	**Precipitantes** Conveliemze indequação sobr regime medicamento; urémios, hospitallizados, individuas e com maior tempo de diagnosico o da Melletuts.
**Reforráçores** Ingestão alimentar iddequau;	Dialisato sem glicose; e filtro dialisalo fluxo. jejum total.

Fonte: adaptado#[9].

Os antecedentes clínicos também foram subdivididos em fatores de risco, condição associada e população em risco, os quais foram listados na Tabela 1.

Dessa forma, foram identificados em sete fatores de risco, sete condições associadas e quatro populações em risco para o DE “Risco de autogestão ineficaz do padrão de glicemia” em pacientes em HD e foram categorizados conforme o MAR em estímulos focais, contextuais e residuais.

Assim, foram identificados como estímulos focais os fatores de risco ingestão alimentar inadequada, perda de peso e jejum total. Quanto aos estímulos contextuais, foram identificados nos seguintes fatores de risco: monitorização inadequada da glicemia durante e/ou após a HD; conhecimento inadequado sobre regime medicamentoso; conhecimento inadequado sobre sinais e sintomas de hipoglicemia; Índice de Massa Corporal alto; nas condições associadas: DM; dialisato sem glicose; resistência à insulina; desnutrição; filtro dialisador de alto fluxo; hiperlipidemia, infecções; e nas populações de risco: indivíduos urêmicos e hospitalizados. Já os estímulos residuais foram identificados nas populações de risco, ou seja, indivíduos idosos e indivíduos com maior tempo de diagnóstico de DM.

**Table 1:** Fatores de risco, condições associadas e população em risco

Fatores de risco	n*	%
Monitorização inadequada da glicemia durante e/ou após a hemodiálise	09	32,2
Conhecimento inadequado sobre regime medicamentoso	08	28,6
Ingestão alimentar inadequada	05	17,8
Perda de peso	01	03,6
Conhecimento inadequado sobre sinais e sintomas de hipoglicemia	01	03,6
Índice de Massa Corporal alto	01	02,7
Jejum total	01	03,6
**Condições associadas**	**N**	**%**
Diabetes Mellitus	19	51,3
Dialisato sem glicose	09	24,3
Resistência à insulina	04	10,8
Desnutrição	03	10,7
Filtro dialisador de alto fluxo	03	08,1
Hiperlipidemia	01	01,1
Infecções	01	02,7
**População em risco**	**N**	**%**
Indivíduos idosos	04	44,4
Indivíduos com maior tempo de diagnóstico de Diabetes Mellitus	01	11,1
Indivíduos urêmicos	01	11,1
Indivíduos hospitalizados	01	11,1

*Mais de um dos antecedentes estava no mesmo estudo, o que justifica a soma superior a 100,0%.

### Elaboração de um esquema pictorial

A TMA desenvolvida neste estudo foi intitulada “Teoria de enfermagem para ‘Risco de autogestão ineficaz do padrão de glicemia’ em pacientes submetidos à HD”, e a partir do agrupamento dos elementos identificados, foi possível construir um pictograma capaz sintetizar a teoria.

A Figura 3 apresenta o pictograma construído para a teoria e seus elementos, explicando as relações entre os conceitos que envolvem o risco de autogestão ineficaz do padrão de glicemia em pacientes adultos e idosos submetidos à HD.

**Figura 3:** Pictograma com os principais conceitos inter-relacionados da teoria de médio “Risco de autogestão ineficaz do padrão de glicemia” em pacientes adultos e idosos submetidos à hemodiálise, Natal, Rio Grande do Norte, Brasil, 2024

Assim, a imagem do ser humano representa o indivíduo submetido à HD com maior risco à variação da glicemia conectado, a partir do acesso vascular, ao dialisador (local de interação dos circuitos de sangue e de solução de diálise e onde ocorre o movimento das moléculas entre a solução de diálise e o sangue através de uma membrana semipermeável) e ao equipo de efluxo (que conduz o sangue do dialisador de volta ao acesso vascular).

Destarte, para explicar o risco de autogestão ineficaz do padrão de glicemia em pacientes submetidos à HD, observa-se que este é influenciado por diversos estímulos que podem provocar a ocorrência de um comportamento ineficaz, como episódios de hipo/hiperglicemia durante e após o tratamento. Esses estímulos (focais, contextuais e residuais) são representados na imagem pelas setas na mesma tonalidade da imagem do indivíduo, influenciando, de forma direta e indireta, o paciente em HD, e são responsáveis por aumentar o risco desse paciente de desenvolver a variação da glicemia durante e após o tratamento.

Dessa forma, observam-se fatores que contribuem para a vulnerabilidade a desequilíbrio no padrão glicêmico e que esses interagem entre si, podendo ser alvo de intervenção independente por parte da equipe de enfermagem. Além disso, as setas em ciclo denotam que os estímulos podem influenciar os indivíduos com diferentes graus de intensidade.

### Construção das proposições da teoria de médio alcance

Para a TMA desenvolvida neste estudo, foram criadas seis proposições, que fornecem as relações de causalidade entre os antecedentes clínicos e atributos essenciais do risco de autogestão ineficaz do padrão de glicemia em pacientes adultos/idosos submetidos à HD.

1. A variação da glicose do paciente submetido à HD é afetada por estímulos oriundos do próprio paciente (idade, comorbidades e autogestão ineficaz) e influenciados pelas ações dos profissionais de saúde atuantes na HD (oferta de alimentação, administração de antiglicemiantes antes da HD, monitorização da glicemia durante e após HD, prescrição de tratamento com dialisadores de alto fluxo, e dialisato sem glicose);
2. Indivíduos em jejum total ou com ingesta alimentar inadequada quando submetidos à HD têm maior risco de glicemia instável;
3. A perda de peso, desnutrição, resistência à insulina, hiperlipidemia, DM, infecções e Índice de Massa Corporal alto em pacientes em HD aumentam o risco de glicemia instável;
4. Indivíduos com maior tempo de diagnóstico da DM, idosos, com uremia e/ou hospitalizados, quando submetidos à HD, podem ser suscetíveis ao risco de glicemia instável;
5. A falta de conhecimento dos pacientes em HD quanto aos sinais e sintomas da hipoglicemia e sobre o regime medicamentoso aumenta o risco de glicemia instável;
6. A não monitorização da glicose do paciente e a monitorização de forma inadequada podem dificultar a identificação do distúrbio glicêmico induzido por HD.

### Estabelecimento das relações de causalidade para a prática

O paciente submetido à HD tem maior risco de autogestão ineficaz do padrão de glicemia, com episódios de hipoglicemia atribuídos a vários fatores, que incluem, devido à doença renal, diminuição da gliconeogênese renal, comprometimento da depuração da insulina pelos rins, degradação deficiente da insulina devido à uremia, aumento da captação de glicose pelos eritrócitos durante a HD, comprometimento das respostas hormonais contra regulatórias, ingesta alimentar inadequada, uso de medicamentos antidiabéticos orais ou insulina exógena sem monitoramento adequado dos níveis de glicose sanguínea, efeitos do tratamento de diálise, e até mesmo idade ou hospitalização^(1,4,5)^.

Além disso, como a secreção de insulina pode ser estimulada pela glicose do dialisato, os pacientes com níveis moderados de função das células β apresentam um risco maior de hipoglicemia com a HD, particularmente após apresentarem grandes excursões hiperglicêmicas. Por outro lado, pacientes com função diminuída ou inexistente das células β apresentam maior risco de hiperglicemia pós-prandial após HD, devido ao aumento da resistência à insulina, adsorção de insulina pelos dialisadores e secreção de hormônios contrarreguladores em resposta ao estado hipoglicêmico^(1,4,5)^.

Conforme a literatura^20^, as relações causais podem ser de sete tipos diferentes: efeito linear; efeito gatilho; efeito de *feedback*; efeito dominó; efeito borboleta; efeito de salto de qualidade; e efeito de causas suficientes. Assim, foram estabelecidas as relações de causa e efeito para o risco de autogestão ineficaz do padrão de glicemia em pacientes submetidos à HD:

- Efeito linear: ingestão alimentar inadequada; jejum total; dialisato sem glicose; filtro dialisador de alto fluxo. Destarte, a exposição ao antecedente gera um consequente, que é a autogestão ineficaz do padrão de glicemia;
- Efeito dominó: monitorização inadequada da glicemia durante e/ou após a HD; conhecimento inadequado sobre regime medicamentoso; conhecimento inadequado sobre sinais e sintomas de hipoglicemia. Nesses casos, um antecedente gera um consequente que levará a uma alteração subsequente;
- Efeito salto de qualidade: Índice de Massa Corporal alto; DM; resistência à insulina; desnutrição; hiperlipidemia; infecções. Nessas situações, a exposição constante a um antecedente clínico pode levar a um consequente de maior gravidade.

Assim, diante do exposto, o manejo da glicemia do paciente em HD é um processo complexo. As relações de causalidade e de evidências para a prática permitem a fundamentação teórica para a validação de DE e para a prática clínica do enfermeiro.

## DISCUSSÃO

Para realizar um diagnóstico acurado, é essencial que o enfermeiro possua um entendimento aprofundado dos conceitos-chave relacionados aos DE. Além disso, é necessário interpretar as respostas humanas com precisão, a fim de escolher intervenções apropriadas e avaliar os resultados obtidos^25^Nesse contexto, a taxonomia da NANDA-I possibilita uma linguagem padronizada dos DEs, o que é fundamental para promover clareza e a consistência na comunicação entre os profissionais de enfermagem, além de fortalecer sua prática e promover o planejamento de cuidados consistentes^8^.

Além disso, essa uniformidade na linguagem contribui para as pesquisas na área da enfermagem, permitindo a comparação e análise de dados em diferentes contextos e regiões geográficas, o que acrescenta informações e suscita discussões no escopo da enfermagem. Dessa forma, fomenta o desenvolvimento e refinamento dos DEs, tornando sua linguagem mais sólida, padronizada e baseada em evidências^8^.

Portanto, em relação ao aperfeiçoamento da taxonomia da NANDA-I, o presente estudo possibilitou o desenvolvimento da teoria de enfermagem para o DE “Risco de autogestão ineficaz do padrão de glicemia” em pacientes submetidos à HD, e a realização da *scoping review* permitiu a compreensão de um gradiente teórico sobre o fenômeno estudado.

Ademais, observou-se a predominância de produções em formato de artigos, o que pode estar associado à rapidez na elaboração e disseminação desse formato, além de ter maior visibilidade, em comparação com dissertações e teses^26^Apesar da notória importância do tema na população estudada, evidenciou-se um quantitativo pequeno de estudos que abordassem o fenômeno da glicemia instável em pacientes submetidos à HD, com predomínio das produções sobre a temática nos Estados Unidos da América. Tal resultado pode ser reflexo do expressivo crescimento no número de casos do DM, principal patologia que leva à doença renal crônica terminal e necessidade de HD^27^.

Entretanto, verificou-se o aumento de publicações nos últimos cinco anos, fato que coaduna com a relevância do tema, uma vez que as alterações nos níveis de glicose são preocupantes e podem desencadear complicações severas. Tanto os picos nos índices glicêmicos quanto sua redução abrupta devem ser evitados e controlados para melhorar a qualidade da terapia, o prognóstico e a segurança do paciente^2^.

É importante destacar que não foram encontrados na revisão realizada estudos que abordassem a temática do DE “Risco de autogestão ineficaz do padrão de glicemia” em pacientes submetidos à HD e o desenvolvimento de uma TMA. Com relação a trabalhos/diretrizes baseados em evidências disponíveis para alvos glicêmicos em pacientes submetidos à HD, a literatura^27^ recomenda que pacientes com DM e em diálise utilizem esquemas de insulina baseados no horário da HD e nos níveis de glicemia pré e pós-diálise, sendo necessária a redução de pelo menos 25% da dose de insulina rápida ou ultrarrápida aplicada logo antes da refeição anterior à diálise, além da manutenção de níveis glicêmicos entre 126 e 200 mg/dL antes e durante a HD.

Outrossim, ao realizar a comparação entre os elementos do DE “Risco de autogestão ineficaz do padrão de glicemia” presente na NANDA-I^8^, que atualmente conta com 28 fatores de risco, 23 condições associadas e 13 populações em risco, a partir da *scoping review,* esse DE em pacientes submetidos à HD apresentou sete fatores de risco, e entre eles, o conhecimento inadequado sobre sinais e sintomas de hipoglicemia se diferencia.

Destaca-se que o paciente necessita conhecer sinais e sintomas da labilidade glicêmica, pois, por falta de conhecimento, a hipoglicemia pode ser diagnosticada erroneamente como hipovolemia, desequilíbrio devido à HD ou devido a desequilíbrios eletrolíticos. Destaca-se que os sintomas de hipoglicemia são divididos em sintomas autonômicos e sintomas de hipoglicemia neurológica. Os sintomas do sistema nervoso autônomo incluem fome, sudorese, ansiedade, parestesia, palpitações, tremores, palidez e taquicardia. E os sintomas da hipoglicemia neurológica incluem fraqueza, tontura e dor de cabeça, confusão, comportamento anormal, comprometimento cognitivo, visão turva, diplopia, cegueira central, hipotermia, epilepsia, e coma^(28, 29^.

Com relação às condições associadas, apenas sete foram elencadas, com destaque para duas delas: dialisato sem glicose e filtro dialisador de alto fluxo. O dialisato é a solução de diálise, que deve ser preparada com água purificada (tratada) e concentrados (eletrólitos necessários para obter a composição prescrita da solução de diálise). Dessa forma, um dialisato sem glicose pode induzir perdas de até 30 g de glicose por sessão, devido à perda da glicose através das membranas capilares, o que pode provocar hipoglicemia^(1, 30^).

Já o filtro dialisador é o local de interação dos circuitos de sangue e de solução de diálise, ou seja, onde ocorre o movimento das moléculas entre a solução de diálise e o sangue através de uma membrana semipermeável^1^Assim, a remoção de substâncias é influenciada por fatores como coeficiente de reflexão, estrutura do poro, permeabilidade hidráulica, superfície da membrana dialisadora e espessura da fibra do filtro dialisador^31^Destarte, as membranas com alta permeabilidade à água e com poros grandes (alto fluxo) apresentam resistência muito baixa e facilitam a difusão das moléculas, o que submete o paciente em uso a maior risco de perda da glicose durante o tratamento^32^.

Já as populações submetidas à HD em risco de glicemia instável identificadas foram os adultos e idosos com maior tempo de diagnóstico de DM, pacientes urêmicos e hospitalizados. Ressalta-se que indivíduos com diagnóstico prévio de DM e em HD têm maior risco à glicemia instável, pois podem estar mais acostumados a um regime de tratamento menos rígido e, portanto, menos propensos a mudar e mais expostos ao risco^33^.

Quanto aos pacientes urêmicos, esses apresentam sinais e sintomas, como náuseas, vômitos, rebaixamento do nível de consciência e desorientação, que podem aumentar o risco de autogestão ineficaz do padrão de glicemia, não somente pela ausência de ingestão de alimentos e de nutrientes, mas também pela necessidade de dietas rígidas com restrição proteica e calórica^34^.

Ademais, a instabilidade glicêmica no paciente hospitalizado pode ocorrer devido a situações de estresse (hiperglicemia do estresse) e pelo desconhecimento prévio do diagnóstico de DM^35^Também são comuns alterações na alimentação devido à internação hospitalar, como a necessidade de jejum prolongado e não aceitação da dieta ofertada. Ademais, pacientes internados com oferta de dieta enteral podem ser suscetíveis à interrupção não programada de administração de alimentação enteral ou parenteral. Além disso, durante a internação, pode ser necessário o uso de medicamentos, como corticosteroides, que podem alterar a taxa de glicemia^36^.

Nesse contexto, com a observação da elevada incidência de variação na glicemia em pacientes submetidos à HD, evidencia-se que aqueles que necessitam dessa terapia apresentam maior suscetibilidade à instabilidade nos níveis de glicose pela própria insuficiência renal, que predispõe a distúrbios na homeostase da glicose em indivíduos com e/ou sem DM, uma vez que o rim desempenha um papel crucial no metabolismo da glicose e da insulina^(1,5,37)^.

Portanto, torna-se imperativo que as intervenções de enfermagem sejam conduzidas com base no julgamento clínico e no conhecimento teórico do enfermeiro^23^, para melhores desfechos dos pacientes em HD, uma vez que essas intervenções têm o potencial de aprimorar o controle glicêmico e, por conseguinte, reduzir as complicações e mortalidade associadas.

### Limitações do estudo

Entre as limitações do estudo, destaca-se que a presente TMA foi construída para os pacientes adultos e idosos submetidos à HD. Assim, a generalização desses resultados para pacientes pediátricos e em outros tipos de TRS deverá ser utilizada com cautela. Outra limitação foi a extração dos conceitos principais, dos atributos essenciais e dos antecedentes clínicos, além de ter sido realizada apenas pela pesquisadora principal, sem o auxílio de outros pesquisadores e/ou de *softwares*.

### Contribuição para área da saúde, enfermagem ou políticas públicas

A realização deste estudo busca ampliar as discussões no meio científico e assistencial acerca da vulnerabilidade do paciente em HD quanto à alteração da glicemia durante o tratamento. E contribui no aprimoramento do DE “Risco de autogestão ineficaz do padrão de glicemia”, que ainda não foi validado para o paciente submetido à HD, o que pode subsidiar a estruturação de estratégias e na reordenação da execução do trabalho da enfermagem, a fim de minimizar a instabilidade da glicemia e seus efeitos no paciente com necessidade desse tratamento.

## CONCLUSÕES

A elaboração teórica identificou dois atributos essenciais: HD e variação glicêmica fora do padrão de normalidade. Foram identificados ainda 18 antecedentes clínicos. Esses foram classificados em fatores etiológicos predisponentes, incapacitantes, precipitantes e reforçadores, subdivididos em sete fatores de risco, sete condições associadas e quatro populações em risco. Outrossim, foram desenvolvidos um pictograma, seis proposições e as relações de causalidade, e evidências para a prática oriundas das inter-relações entre esses conceitos foram classificadas em efeitos linear, dominó e salto de qualidade.

Assim, a TMA do DE “Risco de autogestão ineficaz do padrão de glicemia” em pacientes submetidos à HD foi elaborada, auxiliando na maior compreensão desse fenômeno no meio científico e assistencial. Sugere-se a realização de estudo posteriores voltados para validar, no campo empírico, a presente TMA na população de adultos submetidos à HD.

## DISPONIBILIDADE DE DADOS E MATERIAL

Os dados de pesquisa estão disponíveis no corpo do artigo.
